# Large capillary hemangioma of the temporal bone with a dural tail sign: A case report

**DOI:** 10.3892/ol.2014.2143

**Published:** 2014-05-13

**Authors:** GUANG YANG, CHENGUANG LI, XIN CHEN, YAOHUA LIU, DAYONG HAN, XIN GAO, KEIJI KAWAMOTO, SHIGUANG ZHAO

**Affiliations:** 1 Department of Neurosurgery, The First Affiliated Hospital of Harbin Medical University, Harbin, Heilongjiang, P.R. China; 2 Institute of Brain Science, Harbin Medical University, Harbin, Heilongjiang, P.R. China; 3 Computer, Electrical and Mathematical Sciences and Engineering Division, King Abdullah University of Science and Technology, Thuwal, Saudi Arabia; 4 Department of Neurosurgery, Kansai Medical University, Osaka, Japan

**Keywords:** capillary hemangioma, dural tail sign, intraosseous hemangioma, temporal bone

## Abstract

The present study reports a rare case of large capillary hemangioma of the temporal bone with a dural tail sign. A 57-year-old female presented with pulsatile tinnitus and episodic vertigo associated with a ten-year history of an intermittent faint headache. Magnetic resonance imaging revealed a mass in the right petrous bone, which was hypointense on T1-weighted images and heterogeneously hyperintense on T2-weighted images, and showed a dural tail sign following gadolinium administration. Pre-operatively, this tumor was believed to be a meningioma. During surgery, the vascular tumor was removed by a modified pterional approach. A histopathological examination indicated that the tumor was a capillary hemangioma. Although intraosseous capillary hemangiomas are rare, they most frequently affect the temporal bone. Hemangiomas of the temporal bone may mimic other more common basal tumors. The diagnosis is most often made during surgical resection. The dural tail sign is not specific for meningioma, as it also occurs in other intracranial or extracranial tumors. The treatment of intratemporal hemangiomas is complete surgical excision, with radiotherapy used for unresectable lesions. To the best of our knowledge, the present study is the fourth case of intraosseous intracranial capillary hemangioma, but the largest intratemporal hemangioma to be reported in the literature to date.

## Introduction

Intracranial capillary hemangiomas (ICHs) are rare benign vascular tumors that may occur at birth or in early infancy (1,2). Primary hemangiomas of the skull are also rare, accounting for 0.2% of all benign tumors of the skull and 0.7% of all osseous neoplasms (3). A total of three intraosseous ICH cases have been published in the English literature to date (4–6). The frontal and parietal bones have been reported to be the most common sites of involvement in the skull, while temporal bone involvement is extremely rare (7). Facial paralysis and hemifacial spasms are common presentations for intratemporal hemangiomas, however, auditory and vestibular dysfunction may also result from these lesions (8). Intratemporal hemangiomas mimic other more common skull base lesions, which makes them difficult to diagnose pre-operatively (9). The current study presents a case of a large capillary hemangioma of the temporal bone with a dural tail sign. This case was believed to be a meningioma pre-operatively due to a dural tail sign and the lack of classical symptoms. Patient provided written informed consent.

## Case report

**Figure 1:**
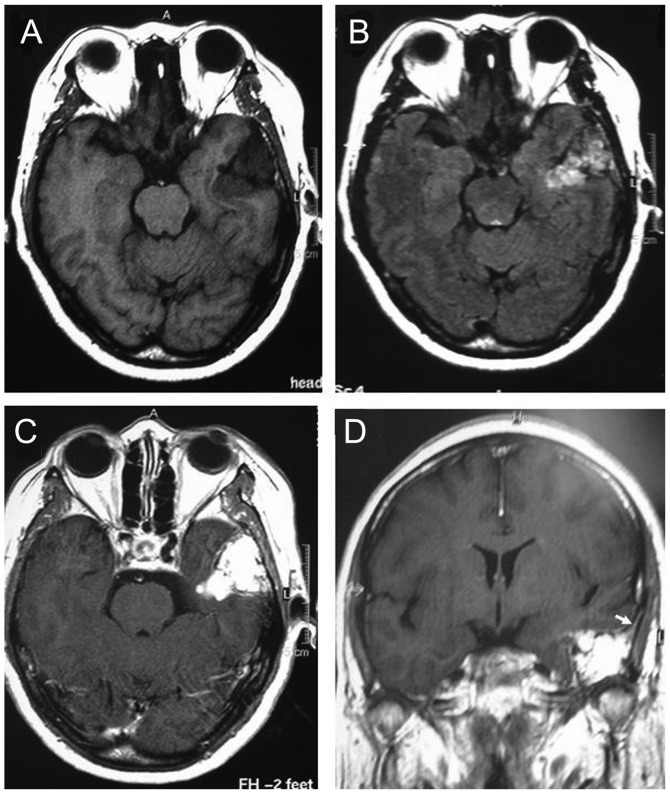
Magnetic resonance imaging prior to surgery. (A) Axial T1-weighted MRI revealing a hypointense lesion in the right petrous bone. (B) Axial T2-weighted magnetic resonance imaging showing the lesion to be hyperintense. (C) Axial and (D) coronal gadolinium-enhanced T1-weighted magnetic resonance imaging showing a heterogeneously enhanced lesion, with a dural tail sign (arrows).

A 57-year-old female presented with pulsatile tinnitus and episodic vertigo associated with a ten-year history of intermittent faint headaches. There was no history of facial twitching or weakness. There was no evidence of facial nerve dysfunction upon physical examination. Pure tone audiometry revealed a hearing level of 28 dB on average in the right ear and a normal hearing level on the left. There was no history of trauma or neurological disturbances. Gadolinium-enhanced magnetic resonance imaging showed a mass measuring 42×36×35 mm in the right petrous bone, which was hypointense on T1-weighted images and heterogeneously hyperintense on T2-weighted images. Additionally, a dural tail sign was shown following gadopentetate dimeglumine administration (Fig. 1).

Surgery was performed using a modified pterional approach. Abnormal vascular soft tissue was identified in the skull base, with skull invasion and involvement of the dura. The invaded temporal bone had cavernous blood-filled spaces within the bony trabeculae. The adjacent dura mater was involved with thickening, but remained intact. The tumor did not affect the bony walls of the horizontal semicircular canal and the facial nerve canal. The patient’s post-operative course was uneventful, and the tinnitus and vertigo disappeared completely in the ensuing weeks. The symptoms did not recur, even after one year of follow-up examinations.

**Figure 2:**
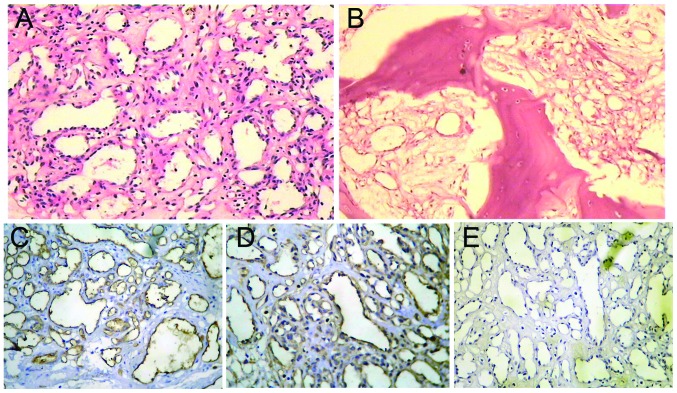
Microscopic and immunohistochemical findings of the tumor. (A) Photomicrographs of the extraosseous fraction of the lesion showing numerous vascular channels and spaces (hematoxylin and eosin; original magnification, ×200) (B) Intraosseous fraction showing multiple tiny fragments of trabecular bone and irregularly-shaped marrow spaces being replaced by thin-walled capillary hemangioma (hematoxylin and eosin; original magnification, ×200). Immunohistochemical analysis showing that the tumor was positive for (C) cluster of differentiation 31 and (D) vimentin, but negative for (E) epithelial membrane antigen (original magnification, ×200).

The histological examination revealed aggregates of primarily capillary-sized microvessels in a vaguely lobular arrangement, with diffusely fibrosed intrabecular spaces containing numerous proliferated, partially small and dilated, thin-walled blood vessels. Factor VIII, vimentin and cluster of differentiation 31 was detected in the blood vessel walls by immunohistochemistry, while epithelial membrane antigen was not (Fig. 2). These findings were consistent with an intraosseous capillary hemangioma.

## Discussion

Intraosseous hemangioma most commonly affects the vertebral column or skull (3,10), but rarely involves the temporal bone. To the best of our knowledge, the present study is the fourth case of intraosseous ICH to date. The majority of the intratemporal vascular tumors reported in the literature have been small (11–13), although Fierek *et al* (7) reported the case of a large 32-mm intratemporal hemangioma. In the present study, the tumor size was larger than any other intratemporal hemangiomas described in the previously published literature.

Histopathology classifies intraosseous hemangiomas into the venous, cavernous and capillary types (14,15). Capillary-type hemangiomas are composed of densely packed loops of fine vessels. Certain studies have described a mixed variety of osseous hemangiomas that contain elements of each of the capillary and cavernous types (16,17). The majority of intraosseous hemangiomas arising from the skull base are cavernous, and only few are capillary (16). Notably, subsequent to reviewing the English-language literature, it may be observed that, although intraosseous capillary hemangiomas are rare, they most frequently affect the skull base (6,7,10,11,18,19), in particular the geniculate ganglion and the fundus of the internal auditory canal, possibly due to the rich vascular network existing around the geniculate fossa and Scarpa’s ganglion (7,16).

Intraosseous hemangiomas are benign tumors that are slow growing and mostly asymptomatic (15). Hemangiomas can cause a variety of symptoms depending on their location and size (14). The characteristic features of intratemporal hemangiomas at the two most frequent sites of occurrence, the geniculate ganglion and the internal auditory canal, include facial nerve paralysis, hemifacial spasms and auditory or vestibular symptoms (10,20,21). Vascular tumors arising in the area of the geniculate ganglion most commonly cause facial paralysis (8). The tumors involving the cochlear otic capsule may cause pulsatile tinnitus or hearing loss (21,22). In the present case, angiomatous erosion of the cochlea or vestibular apparatus was not found, and the symptoms disappeared gradually in the weeks following the surgery; the vertigo and pulsatile tinnitus may have therefore been a result of the compression of these structures.

In the present study, although the hemangioma intruded into the brain tissue, the adjacent dural mater remained intact, revealing no tumorous invasion of the nerve sheath. This is consistent with other previous studies on hemangioma and indicates a compression neuropathy rather than direct invasion (9,18). More commonly however, hemangiomas produce an intense perineural reaction that precludes the establishment of an oncologically sound cleavage plane between the tumor and the affected nerve (16).

Hemangiomas of the temporal bone may mimic other more common cranial base tumors, including acoustic tumors, facial neuromas, meningiomas, cholesteatomas, glomus tumors and metastatic tumors (9,16,23,24). The pre-operative diagnosis of ICH is challenging, and angiography may raise the possibility of a diagnosis (25). In the present study, pre-operatively, meningioma was considered to be the most likely diagnosis, as the lesion showed strong enhancement following gadopentetate dimeglumine administration and had a dural tail sign. However, a dural tail may also be present in association with other intraaxial and extraaxial lesions. Although this sign was highly indicative, it was not specific for the diagnosis of meningioma (26). Direct tumor invasion or reactive meningeal changes may cause the dural tail sign, and it is present in neoplastic and non-neoplastic lesions (27,28). Politi *et al* (27), also described a patient with hemangioma of the frontal bone with a dural tail sign. The case was similar to that of the present patient as the hemangioma was large and the dura mater remained intact. The dural tail may be therefore be attributed to the proliferation of the connective tissue, hypervascularity or vascular dilatation within the dura adjacent to the cranial masses.

The treatment of ICH remains empirical (25). The majority of capillary hemangiomas exhibit a self-limited course and spontaneously regress (1,2,29). However, surgery remains an option for symptomatic ICH, and total resection should be the goal (1). As a complete resection is extremely difficult for hemangiomas of the skull base, a successful excision requires the appropriate surgical approach and technique (16). The preferred modality of treating intratemporal hemangiomas is complete surgical excision, with radiotherapy reserved for unresectable lesions (1,9). Capillary hemangiomas are associated with a high recurrence rate of 43.5% following incomplete resection (1,21). However, in the present patient, a repeat computed tomography scan two years after the surgery revealed no recurrence.

In conclusion, the present study reports the fourth case of intraosseous ICH, but the largest intratemporal hemangioma thus far. ICH may be considered as a likely diagnosis when the tumor involves the skull with a dural tail sign.

## References

[1] Morace R, Marongiu A, Vangelista T, Galasso V, Colonnese C, Giangaspero F, Innocenzi G (2012). Intracranial capillary hemangioma: a description of four cases. World Neurosurg.

[2] Zheng SP, Ju Y, You C (2012). Giant intracranial capillary hemangioma in a 3-year-old child: case report and literature review. Clin Neurol Neurosurg.

[3] Heckl S, Aschoff A, Kunze S (2002). Cavernomas of the skull: review of the literature 1975–2000. Neurosurg Rev.

[4] Shah ZK, Peh WC, Shek TW, Wong JW, Chien EP (2005). Hemangioendothelioma with an epithelioid phenotype arising in hemangioma of the fibula. Skeletal Radiol.

[5] Frei-Jones M, McKinstry RC, Perry A, Leonard JR, Park TS, Rubin JB (2008). Use of thalidomide to diminish growth velocity in a life-threatening congenital intracranial hemangioma. J Neurosurg Pediatr.

[6] Suss RA, Kumar AJ, Dorfman HD, Miller NR, Rosenbaum AE (1984). Capillary hemangioma of the sphenoid bone. Skeletal Radiol.

[7] Fierek O, Laskawi R, Kunze E (2004). Large intraosseous hemangioma of the temporal bone in a child. Ann Otol Rhinol Laryngol.

[8] Friedman O, Neff BA, Willcox TO, Kenyon LC, Sataloff RT (2002). Temporal bone hemangiomas involving the facial nerve. Otol Neurotol.

[9] Glasscock ME, Smith PG, Schwaber MK, Nissen AJ (1984). Clinical aspects of osseous hemangiomas of the skull base. Laryngoscope.

[10] Mangham CA, Carberry JN, Brackmann DE (1981). Management of intratemporal vascular tumors. Laryngoscope.

[11] Lo WW, Horn KL, Carberry JN, Solti-Bohman LG, Wade CT, Brackmann DD, Waluch V (1986). Intratemporal vascular tumors: evaluation with CT. Radiology.

[12] Lo WW, Shelton C, Waluch V, Solti-Bohman LG, Carberry JN, Brackmann DE, Wade CT (1989). Intratemporal vascular tumors: detection with CT and MR imaging. Radiology.

[13] Martin N, Sterkers O, Nahum H (1992). Haemangioma of the petrous bone: MRI. Neuroradiology.

[14] Gottfried ON, Gluf WM, Schmidt MH (2004). Cavernous hemangioma of the skull presenting with subdural hematoma. Case report. Neurosurg Focus.

[15] Reis BL, Carvalho GT, Sousa AA, Freitas WB, Brandão RA (2008). Primary hemangioma of the skull. Arq Neuropsiquiatr.

[16] Liu JK, Burger PC, Harnsberger HR, Couldwell WT (2003). Primary intraosseous skull base cavernous hemangioma: Case report. Skull Base.

[17] Tsao MN, Schwartz ML, Bernstein M, Halliday WC, Lightstone AW, Hamilton MG, Jaywant S, Laperriere N (2003). Capillary hemangioma of the cavernous sinus. Report of two cases. J Neurosurg.

[18] Eby TL, Fisch U, Makek MS (1992). Facial nerve management in temporal bone hemangiomas. Am J Otol.

[19] Hsueh PJ, Chen WY, Chiang YC, Lee FP (2007). Capillary hemangioma of the middle ear. Otolaryngol Head Neck Surg.

[20] Burton L, Burton EM, Welling DB, Marks SD, Binet EF (1997). Hemangioma of the temporal bone in a patient presumed to have Ménière’s syndrome. South Med J.

[21] Tokyol C, Yilmaz MD (2003). Middle ear hemangioma: a case report. Am J Otolaryngol.

[22] Verret DJ, Spencer Cochran C, Defatta RJ, Samy RN (2007). External auditory canal hemangioma: case report. Skull Base.

[23] Malde R, Moss T, Malcolm G, Whittlestone T, Bahl A (2008). Multiple intraosseous calvarial hemangiomas mimicking metastasis from renal cell carcinoma. Adv Urol.

[24] Simon SL, Moonis G, Judkins AR, Scobie J, Burnett MG, Riina HA, Judy KD (2005). Intracranial capillary hemangioma: case report and review of the literature. Surg Neurol.

[25] Mirza B, Shi WY, Phadke R, Holton JL, Turner C, Plant GT, Brew S (2013). Strawberries on the brain - intracranial capillary hemangioma: two case reports and systematic literature review in children and adults. World Neurosurg.

[26] Rokni-Yazdi H, Azmoudeh Ardalan F, Asadzandi Z, Sotoudeh H, Shakiba M, Adibi A, Ayatollahi H, Rahmani M (2009). Pathologic significance of the ‘dural tail sign’. Eur J Radiol.

[27] Politi M, Romeike BF, Papanagiotou P, Nabhan A, Struffert T, Feiden W, Reith W (2005). Intraosseous hemangioma of the skull with dural tail sign: radiologic features with pathologic correlation. AJNR Am J Neuroradiol.

[28] Rokni-Yazdi H, Sotoudeh H (2006). Prevalence of ‘dural tail sign’ in patients with different intracranial pathologies. Eur J Radiol.

[29] Phi JH, Kim SK, Cho A, Kim DG, Paek SH, Park SH, Wang KC (2012). Intracranial capillary hemangioma: extra-axial tumorous lesions closely mimicking meningioma. J Neurooncol.

